# Traditional Foods, Rural Heritage, and Market Resilience

**DOI:** 10.3390/foods15122051

**Published:** 2026-06-06

**Authors:** Luciano Gutierrez, Maria Sabbagh

**Affiliations:** Department of Agricultural Sciences, University of Sassari, 07100 Sassari, Italy; msabbagh@uniss.it

**Keywords:** traditional foods, protected designation of origin, rural heritage, market resilience, willingness to pay

## Abstract

Traditional food systems are increasingly threatened by industrialised agri-food production based on standardised processes, economies of scale, and lower production costs. This transformation risks undermining not only the economic viability of artisanal producers but also the cultural heritage, pastoral knowledge, and territorial identities embedded in traditional foods. This study contributes to rural studies and food heritage research by examining whether consumers’ willingness to pay a premium for traditionally produced foods can sustain endangered rural production systems within competitive PDO markets. Focusing on Fiore Sardo PDO cheese, the study combines a Bertrand duopoly framework with the Theory of Planned Behaviour (TPB) to connect market competition, consumer beliefs, and support for traditional agri-food systems. Data from 1640 Italian consumers were analysed using structural equation modelling. The findings show that attitudes towards cultural preservation, social recognition of traditional production, and perceived support for shepherd communities significantly influence consumers’ willingness to purchase and pay premium prices for traditionally produced cheese. Consumers associate artisanal production not only with superior sensory quality and authenticity but also with the protection of cultural identity, traditional pastoral practices, and rural landscapes. By integrating behavioural and economic perspectives, the study demonstrates that willingness to pay operates as a market mechanism through which consumers actively contribute to the resilience of traditional food systems facing industrial competition. The study advances existing literature by showing how cultural values, behavioural intentions, and market dynamics jointly shape the economic sustainability of traditional foods.

## 1. Introduction

Traditional foods are increasingly recognised for their cultural, nutritional, and socioeconomic value. Within the European Union, schemes such as the Protected Designation of Origin (PDO), Protected Geographical Indication (PGI), and Traditional Speciality Guaranteed (TSG) aim to safeguard and promote foods linked to specific territories, production methods, and cultural practices (EC Reg. No. 1143/2024). Although there is no universally accepted definition of traditional food, most authors agree that these products are rooted in local heritage, craft-based knowledge, and region-specific agricultural systems [1,2]. Their appeal lies not only in sensory qualities but also in their associations with biodiversity, rural identity, and artisanal expertise [3,4,5].

However, traditional agri-food systems are increasingly threatened by globalisation and industrialisation. Food manufacturing has shifted towards large-scale, mechanised production with lower costs and standardised outputs [6,7]. In PDO supply chains, this can create coexistence and tension between small-scale traditional producers and larger industrial firms. Traditional producers face higher labour costs and limited economies of scale, whereas industrial firms can offer PDO-labelled foods at lower prices despite using standardised, less place-based methods. As a consequence, traditional production may become economically unviable, putting the livelihoods of rural producers and the cultural landscapes they sustain at risk.

Despite the growing literature on traditional foods and geographical indications, an important research problem remains underexplored: whether traditional agri-food systems characterised by higher production costs can remain economically viable when competing with industrial producers operating within the same PDO market. Existing studies have largely focused either on consumer preferences for quality-labelled foods or on the socio-cultural value of traditional products, while paying limited attention to how market competition, consumer perceptions, and behavioural intentions interact to influence the survival of traditional production systems. As a result, there is still a limited understanding of the mechanisms through which consumers may contribute to the economic resilience of rural cultural heritage through their purchasing behaviour.

To address this gap, this study investigates whether consumers’ willingness to buy and pay a premium for traditionally produced PDO foods can sustain traditional rural production systems in competitive markets. Specifically, the study combines a simplified Bertrand duopoly framework with the Theory of Planned Behaviour (TPB) to examine both the economic conditions under which traditional producers can maintain price premiums and the behavioural factors influencing consumers’ support for traditional products.

The purpose of the Bertrand framework in this study is not to estimate market equilibria empirically, but rather to provide a conceptual economic interpretation of the conditions under which traditional producers may sustain price premiums despite higher production costs. In this sense, the model serves as a theoretical complement to the TPB analysis by illustrating why consumers’ willingness to pay is essential for the economic viability of traditional production systems.

Using Fiore Sardo PDO cheese as a case study, the research aims to analyse how attitudes, subjective norms, perceived behavioural control, and beliefs related to cultural heritage and rural identity shape willingness to buy and willingness to pay for traditionally produced cheese.

The Fiore Sardo PDO cheese is produced in Sardinia from the milk of Sardinian sheep. It is a traditional pastoral product deeply rooted in the island’s local identity and rural livelihoods. Fiore Sardo is characterised by a hard, compact texture, a natural smoky aroma from light wood smoking, and an intense, persistent flavour profile that becomes increasingly piquant with maturation. The cheese typically exhibits herbaceous and pastoral notes associated with Sardinian grazing systems, and its sensory attributes are strongly influenced by production methods. The supply chain is characterised by two types of producers: small-scale shepherds who maintain artisanal practices, and larger dairies that industrialise part of the process and access broader markets. Despite sharing the PDO certification, these two production systems differ significantly in cost structures, production methods, and perceived authenticity. Consumer express support for the traditional product is therefore crucial to preserving this rural heritage.

This study advances the literature in three ways. First, it connects economic competition theory and behavioural intention models to assess the economic sustainability of traditional food systems. Second, it demonstrates how attitudes, social norms, and perceived behavioural control influence consumers’ willingness to pay a premium for traditional PDO products. Third, it broadens discussions in rural studies by illustrating how cultural values, identity, and market competition converge to shape the resilience of rural agri-food systems.

The remainder of the paper is structured as follows. Section 2 presents the theoretical framework, including the Bertrand model and the TPB. Section 3 describes the data, survey design, and analytical approach. Section 4 reports the empirical results. Section 5 discusses the implications for rural development, producer strategies, and policy. Section 6 provides conclusions and suggestions for future research.

## 2. Proposed Theoretical Model and Research Hypotheses

### 2.1. A Bertrand Model for Traditional and Industrial Firms

In this section, we analyse the conditions under which traditional agri-food producers can remain competitive against industrial firms in a specific PDO market. We consider two representative firms that produce a homogeneous PDO-labelled product but use different production systems. The traditional firm relies on labour-intensive, small-scale methods, resulting in higher marginal costs but higher perceived quality. The industrial firm uses mechanised production, leading to lower costs but lower perceived quality.

We propose a simplified version of Singh and Vives [8]. Bertrand-like model, where the two firms compete, and each firm tries to set the price $p_{i}$ to maximise its profit $\pi_{i}$:

$$\pi_{i}=(p_{i}-c_{i})\;q_{i}\left(p_{i},p_{j}\right),i\in \left(T,I\right)$$

where we assume constant marginal costs $c_{i}$, and the quantities demanded q depend on their own price $p_{i}$, taking the other price $p_{j}$, with j≠i, as given. The indices *T* and *I* refer to traditional and industrial products, respectively.

To simplify the analysis, we define two linear demand equations: one for the traditional product $q_{T}$ and the other for the industrial product $q_{I}$:

(1) $$\begin{array}{cc} q_{T} & =a_{T}-bp_{T}+dp_{I}\\ q_{I} & =a_{I}-bp_{I}+dp_{T} \end{array}$$

where, for the sake of simplicity, we assume that the two firms have the same slope b>0 and the same $d\in \left(0,b\right)$, i.e., the two products are substitutes. Now, define the quality of the two products as $v_{T}\;\mathrm{and}\;v_{I}$, such that the two intercepts $a_{i}$ in the inverse demand equations become $a_{T}=a+kv_{T}$ and $a_{I}=a+kv_{I}$ with k>0

Substituting in (1), computing the first-order conditions, i.e., $\partial \pi_{i}/\partial p_{i}=0$ for $i\in \left\{T,I\right\}$, and solving for $p_{i}$, we obtain:

$$\begin{array}{cc} p_{T}^{*} & =\frac{da_{I}+2ba_{T}+2b^{2}c_{T}+bdc_{I}}{4b^{2}-d^{2}}\\ p_{I}^{*} & =\frac{da_{T}+2ba_{I}+2b^{2}c_{I}+bdc_{T}}{4b^{2}-d^{2}} \end{array}$$

Note that the second-order conditions are easily satisfied, $\partial^{2}\pi_{i}/\partial p_{i}^{2}=-2b<0$ for $i\in \left(T,I\right)$, and the existence of the equilibrium requires that $\left(4b^{2}-d^{2}\right)>0$, i.e., 2b>d, which implies that the two products $\left(T,I\right)$ are not too close substitutes or, in other terms, each firm’s optimal price does not overreact to the rival’s price.

We can now examine the conditions under which a price differential exists between the traditional and industrial products:

$$p_{T}-p_{I}=\frac{\left(a_{T}-a_{i}\right)+b\left(c_{T}-c_{I}\right)}{2b+d}$$

Given that $\left(2b+d\right)>0,$ the price of a traditionally made product will be higher than that of a product produced using industrial processes if $\left(a_{T}-a_{I}\right)>0$, which is the case if the quality of a traditionally made product is perceived as higher than that of an industrial product, i.e., $(v_{T}-v_{I})>0,$ and $(c_{T}-c_{I})>0,$ i.e., the marginal cost of a traditional firm is higher than that of the industrial firm.

Thus, the Bertrand model suggests, in case $(c_{T}-c_{I})>0$ holds, the traditional firm can sustain a price premium even in the presence of competition. This outcome depends on consumers recognising and valuing the higher quality associated with traditional production. In other words, the economic viability of traditional foods requires behavioural acceptance, not just cost-based competitiveness, underscoring the importance of understanding consumer attitudes and willingness to pay a premium for traditional products. In the following, we briefly introduce the Theory of Planned Behaviour model [9], which will help us determine whether Bertrand’s conditions may hold for our case study on the Fiore Sardo PDO cheese.

### 2.2. The Theory of Planned Behaviour

The TPB ()[9] is a widely used behavioural model that extends the theory of reasoned action [10]. The idea of reasoned action proposes that behavioural intention (e.g., “I intend to consume traditionally made PDO products”) mediates the link between attitude and actual behaviour. Furthermore, [10] suggested that social norms influence intention predictions. Attitude involves assessing behavioural outcomes as positive or negative, good or bad, pleasant or unpleasant (e.g., “For me, buying traditionally made PDO products would be good”). Subjective norms, however, refer to social pressure from significant individuals, such as family members (like a spouse, children, or parents), neighbours, or colleagues whose opinions matter for many actions (such as “Most of the important people in my life believe that I should eat traditionally made PDO products”). The TPB adds a third concept, perceived behavioural control, as a predictor of intention. According to [9], perceived behavioural control is a person’s ability to perform a specific behaviour based on non-motivational factors such as access to resources/technology, time, skills, money, others’ cooperation, and knowledge (for example, “For me, buying traditionally made PDO products would be easy”). Therefore, TPB offers insights into various psychosocial factors influencing the intention to consume and actual behaviour (see Armitage and Conner [11] for a review).

Only a few studies have employed the TPB approach to investigate consumer intentions and the willingness to pay for traditional and industrial products, as well as their social-psychological determinants. However, they focused primarily on the relationship between a healthy diet and the consumption of traditional or industrial food products. For instance, Hojjati and Mirzaei [12] used TPB to explore consumer behaviour in choosing industrial dairy products over traditional alternatives. They found that attitudes and subjective norms have a significant, positive influence on the intention to consume industrial dairy products rather than traditional ones. Notably, health consciousness was identified as the strongest predictor of this behavioural intention. Otherwise, Sogari and Pucci [13] found that a positive attitude toward traditional eating enhances consumers’ intention to adopt a healthy diet.

**Figure 1:**
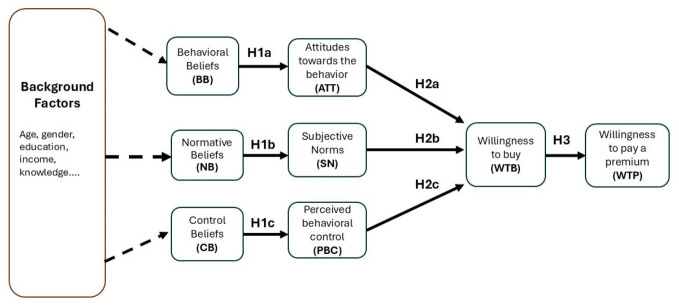
Conceptual framework.

This study contributes to the literature by identifying the behavioural factors, beliefs, attitudes, subjective norms, and perceived behavioural control that explain why consumers may prefer traditionally produced goods over industrial alternatives in markets protected under the PDO scheme, which assures the quality of specific products. The conceptual framework, presented in Figure 1, is grounded in the Theory of Planned Behaviour (TPB). According to Ajzen (1991), intention reflects an individual’s readiness to perform a specific behaviour. Here, the willingness to buy (WTB) a traditionally made, PDO-labelled product is posited as the immediate antecedent of actual purchasing behaviour, as reflected in the willingness to pay a premium for traditional cheese. Within the TPB framework, attitude (ATT), subjective norm (SN), and perceived behavioural control (PBC) represent the key antecedents of WTB. These direct predictors are shaped by underlying beliefs: behavioural beliefs (BB) regarding expected outcomes, normative beliefs (NB) concerning social expectations, and control beliefs (CB) about factors that may enable or constrain the behaviour.

Examining the associations between these belief structures and their corresponding predictors (i.e., BB–ATT, NB–SN, and CB–PBC) provides valuable insights for designing targeted interventions. Such interventions can help sustain traditional production systems within a PDO labelled market by addressing salient beliefs that shape ATT, SN, and PBC, thereby enhancing consumers’ WTB and WTP.

According to the TPB framework, beliefs represent the cognitive foundations of behavioural intentions because they shape individuals’ evaluations of a behaviour, perceptions of social expectations, and perceived ability to perform the behaviour [9,14]. In the context of traditional foods, consumers who believe that artisanal production supports rural livelihoods, preserves authenticity, and protects cultural heritage are more likely to develop favourable attitudes towards purchasing these products. Similarly, perceptions regarding the expectations and behaviours of significant others contribute to subjective norms, while beliefs about product accessibility and affordability influence perceived behavioural control.

Therefore, we hypothesise that:

**H1.**  *Behavioural beliefs (H1a), normative beliefs (H1b), and control beliefs (H1c) all positively influence perceived behavioural control (PBC), attitudes (ATT), and subjective norms (SN) towards purchasing traditionally made PDO products.*

TPB posits that attitudes, subjective norms, and perceived behavioural control are the primary antecedents of behavioural intention [9]. In food consumption contexts, previous studies have shown that positive attitudes towards authenticity and tradition, social approval from peers and family, and perceived ease of accessing traditional products significantly influence consumers’ purchase intentions [11,15]. Therefore, consumers who positively evaluate traditionally produced PDO foods, perceive social support for their consumption, and feel capable of purchasing them are expected to show a stronger willingness to buy.

We hypothesise that:

**H2.**  *Attitudes (ATT) (H2a), subjective norms (SN) (H2b), and perceived behavioural control (PBC) (H2c) are all predictors of the willingness to buy (WTB) a traditionally made PDO product. The PBC refers to an individual’s belief in their own ability to perform a specific behaviour. The ATT indicates whether the individual holds a positive or negative view of that behaviour. SN reflects perceived social pressure, that is, what others think about whether one should engage in that behaviour.*

Within TPB, behavioural intention is considered the immediate antecedent of actual behaviour and behavioural commitment [9]. In contingent valuation and food choice studies, willingness to pay is frequently interpreted as a monetary manifestation of behavioural intention [16]. Consequently, consumers who are more willing to buy traditionally produced cheese are expected to be more willing to pay price premiums for these products.

The final hypothesis is:

**H3.**  *The WTB a traditionally made cheese positively impacts the WTP a premium price for a traditionally made cheese.*

## 3. Materials and Methods

### 3.1. Data Collection and Sample

Data were collected in September 2025 using an online survey (Qualtrics^®^), USA administered by the Bilendi, Franceagency. A stratified sample of 313 Sardinians and 1339 residents of other Italian regions was selected based on age, gender, income, and education. Participants first provided sociodemographic characteristics and household cheese consumption. The samples were representative of both regional and national populations, stratified by age, education, and income.

This study was a non-interventional, anonymous, cross-sectional survey of adult participants posing no more than minimal risk. Respondents were recruited from the established online research panel of Bilendi, a professional market and opinion research agency. Panel enrolment, recruitment, and the administration of informed consent were carried out by the agency in accordance with its GDPR-compliant data governance procedures and applicable professional research codes. Panel members participate voluntarily, having given prior consent to receive survey invitations, and may decline any individual survey or withdraw at any time without consequence. For the present study, respondents were additionally informed of its specific purpose, the confidentiality of their data, and their right to withdraw, and provided informed consent digitally prior to participation. The research team received only anonymised response data and had no access to information identifying individual participants. The survey collected no special-category personal data within the meaning of Article 9 of the EU General Data Protection Regulation, and responses were anonymous, such that no individual could be identified (GDPR Recital 26). In accordance with national and institutional regulations, formal ethics committee approval was not required. The study was nonetheless conducted in accordance with the ethical principles of the Declaration of Helsinki.

The questionnaire was preliminarily piloted with a sample of 300 respondents to define the willingness-to-pay bid range and to verify the clarity, reliability, and overall comprehensibility of the survey instrument prior to data collection.

### 3.2. The Questionnaire

#### 3.2.1. Questionnaire, TPB Measures, and Eliciting WTP

A structured questionnaire comprising three main sections was developed following a thorough review of the literature, primarily utilising measures adapted from previous studies. The first section collected sociodemographic information from participants, including age, gender, income, employment status, and household size. We then checked the household’s cheese consumption. If there was no consumption, the interview was concluded. We proceed with the interview by asking about the average monthly consumption and expenditure on cheese. We also inquired about preferences for cheese texture, categorising responses into three groups: soft, semi-hard, and hard. Additionally, we inquired about cheese ageing preferences, categorising options by ageing period, from fresh cheese to those aged for more than six months. A question was included to determine preferences for quality labels: Protected Designation of Origin (PDO), Protected Geographical Indication (PGI), and Traditional Speciality Guaranteed (TSG). Finally, we asked respondents to share their preferences regarding specific cheese characteristics they consider when purchasing cheese, such as ease of obtaining product information, price level, geographical origin, brand, and cheese awards. The respondent can choose from a seven-point scale, ranging from “definitely irrelevant” to “definitely important.”

After presenting the Fiore Sardo PDO cheese to participants, we asked whether they had previously tasted it. Only those who answered yes were allowed to proceed to the next sections of the questionnaire.

Only participants who had previously tasted Fiore Sardo PDO cheese were included in the empirical analysis. This sampling choice was intentional and theoretically grounded in the Theory of Planned Behaviour [9], according to which prior experience contributes to the formation of behavioural beliefs, attitudes, subjective norms, and perceived behavioural control. Evaluating willingness to pay for traditionally produced Fiore Sardo cheese requires that respondents have at least minimal familiarity with the product and its sensory and cultural characteristics. Consumers without prior consumption experience may be unable to meaningfully distinguish between traditional and industrial production methods or evaluate authenticity-related attributes. Therefore, the study focuses specifically on consumers with direct product experience to analyse the behavioural mechanisms associated with support for traditional production systems. In synthesis, the sample was purposefully restricted to experienced consumers because the research question concerns evaluative behavioural mechanisms rather than market penetration. Nevertheless, this choice limits the generalisability of the findings to the broader population, and future research could investigate how unfamiliarity, lack of awareness, or product rejection influence barriers to market expansion.

The second section included questions designed to evaluate belief constructs (BB, NB, and CB); the third section contained statements aimed at exploring constructs (ATT, SN, and PBC); and the final section featured questions to assess willingness to pay a premium for Fiore Sardo PDO cheese produced using traditional methods. These constructs are based on prior applications [17,18,19] and have been defined through the methodological considerations of [14]. The items in each construct, BN, NB, and CB, were anchored on a unipolar seven-point scale from “extremely unlikely” to “extremely likely,” following the suggestion of [20]. The constructs ATT, SN, PBC and WTB were also anchored on a unipolar seven-point scale from “strongly disagree” to “strongly agree”. In the Measurement model section we list the constructs and items used in this study, including the distribution across the seven-point scales, means, and standard deviations for the two samples. To assess the monetary value attributed to traditional cheese, participants were asked about their willingness to pay a higher price for one kilogram of Fiore Sardo PDO-labelled, traditionally produced cheese compared to one kilogram of Fiore Sardo PDO-labelled, industrially produced cheese. A payment card technique was employed for this purpose. Participants were presented with a range of prices from 0.0 euros to 16 euros or more per kilogram and asked to select the highest premium they were willing to pay. Finally, participants were informed that purchasing more expensive, traditionally made Fiore Sardo PDO cheese would deplete their limited budget, reducing their capacity to buy other items.

#### 3.2.2. Data Analysis

Partial Least Squares Structural Equation Modelling (PLS-SEM) was employed to examine the hypothesised model using SmartPLS4 4 [21]. This analytical approach adopts a causal–predictive perspective to structural equation modelling (SEM). PLS-SEM was selected over covariance-based SEM due to the study’s primary emphasis on the explanatory capacity of the latent constructs, rather than on reproducing the covariance matrix to achieve overall model fit.

In the data analysis phase, indicator loadings and internal consistency reliability were assessed, with reliability evaluated through the composite reliability coefficient [22]. The convergent validity of the measurement model was subsequently examined using Average Variance Extracted (AVE), followed by evaluation of the structural model’s validity. Multicollinearity was assessed to ensure that it did not distort the regression estimates by calculating the Variance Inflation Factor (VIF). The HeteroTrait-MonoTrait ratio indicator (HTMT) has been used for further analysis of the discriminant validity of each construct [23]. The adequacy of the structural model was further evaluated using the Standardised Root Mean Square Residual (SRMR). In addition, the adjusted coefficient of determination (R^2^) for the endogenous constructs was reported [24].

Since PLS-SEM is a distribution-free estimation method, statistical inference was performed using a bootstrapping procedure with 10,000 resamples, employing bias-corrected confidence intervals [25] and a two-tailed test at the 5% significance level, as recommended by Streukens and Leroi-Werelds [26]. We finally calculate the Q^2^ values for the PLS-SEM models. The statistics indicate the model’s out-of-sample predictive power.

## 4. Results

The demographic and socioeconomic characteristics of the participants are shown in Table 1. Most participants were female (55% in Sardinia and 52% in Other Italian Regions (OIR)), with ages ranging from 35 to 64 years. Educational levels were classified as lower high school diploma, high school diploma, or university degree. Results showed that most participants in Sardinia held a high school diploma and had an annual income between 20,000 and 39,000 euros, with higher incomes observed in OIR. The average family size was fewer than three members. More than 60% of Sardinian respondents were employees, while under 10% were self-employed. In OIR, these figures changed to 50% and 11%, respectively. Before asking specific questions about cheese consumption, we first inquired about cheese intake in respondents’ families. In Sardinia, 4% reported not consuming cheese, whereas in OIR, this figure was 2%. The survey was terminated for respondents who did not report consuming cheese.

Respondents in OIR spent more on cheese than those in Sardinia. However, in percentage terms, the total expenditure and consumption of pecorino cheese were higher in Sardinian regions. The results also indicate that both Sardinian and OIR consumers strongly prefer semi-hard cheeses, with 80% in both groups choosing this option. However, some differences emerge in their preferences for other categories. Sardinian respondents show slightly less interest in soft cheeses (48%) and hard cheeses (43%) compared to consumers from other Italian regions (50% and 52%, respectively). Regarding ageing, fresh and lightly aged cheeses (under 30 days) are slightly less preferred in Sardinia (44–37%) than elsewhere in Italy (55–45%). Preferences again align for medium- to longer-ageing periods (1–6 months), with similar figures across groups, although consumers from other regions tend to have a marginally higher appreciation for these maturations. Overall, while both groups favour semi-hard cheeses, consumers outside Sardinia demonstrate a slightly stronger inclination towards harder and fresher cheeses.

A question was included to assess preferences for quality labels: PDO, PGI, and TSG. Among Sardinian respondents, 39% expressed a preference for PDO cheeses, followed by 31% for PGI cheeses. The share of PDO cheeses increased among the OIR participants, reaching 63% of respondents. We also asked participants about their cheese purchasing preferences, specifically regarding product details such as information on the characteristics of the cheese, price, origin, brand, and awards. The results (not reported here for brevity) highlight notable differences in how consumers in Sardinia and other Italian regions value cheese traits. Among Sardinian respondents, geolocation is the most decisive factor, with over half indicating it is “definitely important,” followed by a significant proportion who rate it as “important.” Information, brand, and price also attract considerable attention, though to a lesser degree. Conversely, awards are mostly seen as non-influential, with the highest share of respondents rating them as “indifferent.” A similar trend is observed among consumers from other Italian regions, where geolocation and information are still the most valued attributes, while awards remain low in importance. Overall, the findings suggest that both groups prioritise origin and authenticity cues over external recognitions such as awards.

### The Measurement Model

We tested the Theory of Planned Behaviour (TPB) model using responses from participants who reported having tasted Fiore Sardo PDO cheese. Specifically, 79% (N = 249) of participants from Sardinia and 40% (N = 532) from the Other Italian Regions (OIR) had sampled the cheese. As expected, the proportion of individuals who had previously tasted Fiore Sardo PDO cheese was higher in Sardinia compared to the OIR.

As reported in Table 2, Table 3 and Table 4, the frequency distributions of behavioural beliefs and attitudes (Table 2), normative beliefs and subjective norms (Table 3), and control beliefs and perceived behavioural (Table 4), did not show significant differences between the groups of participants living in Sardinia and in OIR. Pairwise mean tests of the null hypothesis that both groups show equal means were not rejected. There is a strong belief that buying cheese produced by traditional methods can improve shepherds’ economic conditions. Additionally, this practice helps preserve the unique flavours of cheese and allows traditional cheeses to be valued higher in monetary terms. The positive beliefs foster favourable attitudes toward the socioeconomic importance of purchasing and consuming traditionally made cheeses. We also noted that positive normative beliefs, reflecting the perception that family members and friends approve of buying traditionally made cheese, can shape both injunctive and descriptive subjective norms. Thus, when individuals believe that others support traditional cheese producers (injunctive norms) and observe that many of their close friends already do so (descriptive norms), they feel a greater social obligation and a greater sense of acceptance to buy traditional cheeses. As a result, these social expectations and observed behaviours combine to create stronger subjective norms, increasing the likelihood that individuals will intend to pay a premium price to purchase traditionally made cheeses. As expected, participants in Sardinia are more likely to believe that finding and buying Fiore Sardo cheese in a shop is easier than those in OIR. This result is particularly important because when individuals believe they can overcome barriers, such as product availability, their perceived behavioural control increases, positively influencing their intention to purchase. Both groups of participants agree that they are willing to buy and pay a higher price for Fiore Sardo PDO cheese produced using traditional methods.

**Figure 2:**
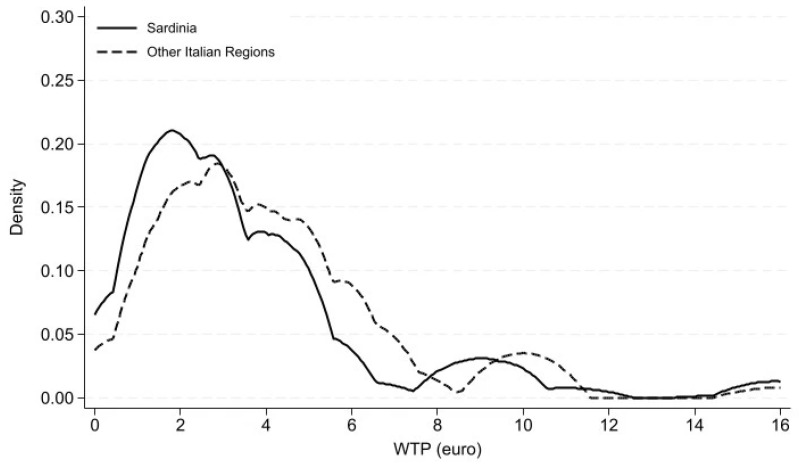
Respondents’ willingness to pay a premium for Fiore Sardo DOP cheese made by shepherds.

Regarding the amount individuals are willing to pay, the study found that the average maximum premium for 1 kg of traditionally made Fiore Sardo pecorino cheese is 3.7 euros (median value 2.5 euros) in Sardinia and 5.5 euros (median value 4.0 euros) in OIR. In Figure 2, we show the estimated distributions of the two participant groups, Sardinia and OIR, using the Epanechnikov kernel. As expected from the mean and median values, the WTP distribution appears right-skewed.

**Table 2:** Attitudes toward buying Fiore Sardo PDO cheese made using traditional methods.

Behavioural Beliefs	Sample	Extremely Unlikely	Moderately Unlikely	Slightly Unlikely	Neither Likely Nor Unlikely	Slightly Likely	Moderately Likely	Extremely Likely	*Me* *an Score*	*Std. Dev.*
		1	2	3	4	5	6	7		
**BB1**: How likely is it that purchasing cheese made using traditional methods, compared to cheese produced by industrial processes, will improve the economic situation of the shepherds?	Sardinia (N: 249)	3.6%	2.0%	4.0%	16.9%	22.5%	23.3%	27.7%	5.33	1.53
	Other Regions (N: 532)	3.2%	2.3%	4.9%	10.1%	19.0%	29.1%	31.4%	5.52	1.51
**BB2**: How likely is cheese produced with traditional methods, compared to a product made with industrial processes, allows for a greater preservation of the typical flavours of cheese?	Sardinia (N: 249)	0.8%	2.0%	3.2%	9.2%	18.5%	21.7%	44.6%	5.86	1.34
	Other Regions (N: 532)	2.1%	2.1%	2.6%	6.2%	16.7%	28.9%	41.4%	5.86	1.38
**BB3**: How likely is it that a consumer attributes a higher monetary value to cheese produced by traditional methods, compared to one produced with industrial processes?	Sardinia (N: 249)	0.8%	2.0%	3.2%	9.2%	18.5%	21.7%	44.6%	5.70	1.24
	Other Regions (N: 532)	0.6%	1.5%	4.3%	7.5%	21.1%	32.7%	32.3%	5.74	1.24
**Attitudes toward the behaviour**	**Sample**	**Strongly disagree**	**Disagree**	**Somewhat disagree**	**Neither agree** **Nor disagree**	**Somewhat Agree**	**Agree**	**Strongly agree**	***Mean Score***	***Std. Dev.***
		−3	−2	−1	0	1	2	3		
**ATT1**: The purchase of Fiore Sardo PDO cheese, produced with traditional methods, is an important economic support for shepherds.	Sardinia (N: 249)	0.0%	0.8%	1.6%	5.6%	22.1%	26.1%	43.8%	2.02	1.08
	Other Regions (N: 532)	0.2%	0.4%	2.1%	7.5%	18.2%	33.1%	38.5%	1.97	1.08
**ATT2**: The purchase of Fiore Sardo PDO cheese, produced using traditional methods, is an important factor in maintaining its typical aromas and flavours.	Sardinia (N: 249)	0.0%	0.4%	0.8%	6.8%	17.7%	27.3%	47.0%	2.12	1.02
	Other Regions (N: 532)	0.2%	0.4%	0.9%	7.1%	15.2%	31.2%	44.9%	2.10	1.03
**ATT3**: The Fiore Sardo PDO cheese, made with traditional methods, must have a higher price per kg than that produced with industrial processes.	Sardinia (N: 249)	0.8%	1.2%	5.2%	8.0%	41.4%	30.9%	12.4%	1.31	1.12
	Other Regions (N: 532)	0.8%	2.3%	3.8%	8.8%	34.4%	36.1%	13.9%	1.37	1.60

**Table 3:** Norms toward buying Fiore Sardo PDO cheese made using traditional methods.

Normative Beliefs	Sample	Extremely Unlikely	Moderately Unlikely	Slightly Unlikely	Neither Likely Nor Unlikely	Slightly Likely	Moderately Likely	Extremely Likely	*Mea* *n Score*	*Std. Dev.*
		−3	−2	−1	0	1	2	3		
**NB1**: How likely is it that the people closest to you (family, friends, etc.) influence the purchase of cheese made with traditional methods, rather than cheese produced with industrial processes?	Sardinia (N: 249)	2.8%	4.4%	8.0%	17.3%	19.7%	29.3%	18.5%	1.08	1.54
	Other Regions (N: 532)	2.1%	4.1%	7.1%	15.6%	26.5%	26.9%	17.7%	1.12	1.45
**NB2**: How likely is it that people like you will also buy cheese made with traditional methods, rather than cheese produced with industrial processes?	Sardinia (N: 249)	0.0%	0.4%	2.0%	7.6%	27.7%	35.3%	26.9%	1.76	1.02
	Other Regions (N: 532)	0.0%	1.1%	2.4%	9.6%	26.1%	35.0%	25.8%	1.69	1.10
**Subjective norms**	**Sample**	**Strongly disagree**	**Disagree**	**Somewhat disagree**	**Neither agree** **nor disagree**	**Somewhat Agree**	**Agree**	**Strongly agree**	***Mean Score***	***Std. Dev.***
		1	2	3	4	5	6	7		
**SN1**: I am interested in the opinions of the people closest to me (family, friends, etc.) about the purchase of Fiore Sardo PDO cheese.	Sardinia (N: 249)	2.4%	6.0%	6.0%	15.7%	24.1%	28.9%	16.9%	5.07	1.51
	Other Regions (N: 532)	1.5%	5.3%	7.1%	12.2%	24.1%	28.8%	21.1%	5.23	1.48
**SN2**: People like me are interested in buying a Fiore Sardo PDO cheese.	Sardinia (N: 249)	0.0%	0.0%	1.2%	7.2%	16.1%	38.6%	36.9%	6.03	0.96
	Other Regions (N: 532)	0.0%	0.0%	2.3%	7.5%	23.3%	42.7%	24.2%	5.79	0.97

**Table 4:** Perceived behavioural control toward buying Fiore Sardo PDO cheese made using traditional methods.

Control Beliefs	Sample	Extremely Unlikely	Moderately Unlikely	Slightly Unlikely	Neither Likely Nor Unlikely	Slightly Likely	Moderately Likely	Extremely Likely	*M* *ean Score*	*Std. Dev.*
		1	2	3	4	5	6	7		
**CB1**: How likely is it that purchasing a Fiore Sardo PDO cheese is easy in the shops in your area?	Sardinia (N: 249)	0.0%	0.4%	0.8%	7.6%	19.7%	30.5%	41.0%	6.02	1.03
	Other Regions (N: 532)	0.4%	1.9%	7.1%	18.8%	24.8%	29.9%	17.1%	5.24	1.28
**CB2**: How likely is it that a higher price for a Fiore Sardo PDO cheese made using traditional methods, compared to cheese produced using industrial processes, will hinder its purchase?	Sardinia (N: 249)	4.8%	7.2%	22.5%	26.5%	16.1%	15.3%	7.6%	4.18	1.54
	Other Regions (N: 532)	4.1%	10.0%	20.7%	19.5%	16.2%	19.0%	10.5%	4.33	1.66
**Perceived behavioural control**	**Sample**	**Strongly disagree**	**Disagree**	**Somewhat disagree**	**Neither agree** **nor disagree**	**Somewhat Agree**	**Agree**	**Strongly agree**	***Mean Score***	***Std. Dev.***
		−3	−2	−1	0	1	2	3		
**PBC1**: If I only wanted to, I could easily buy a Fiore Sardo PDO cheese made using traditional methods.	Sardinia (N: 249)	0.4%	0.4%	2.4%	8.8%	25.7%	32.5%	29.7%	1.76	1.11
	Other Regions (N: 532)	0.2%	2.3%	4.9%	17.7%	29.7%	26.7%	18.6%	1.29	1.24
**PBC2**: I am sure that I could easily recognise, through observation and/or tasting, a Fiore Sardo PDO cheese produced with traditional methods compared to a Fiore Sardo PDO cheese produced with industrial methods.	Sardinia (N: 249)	1.6%	2.4%	4.4%	29.3%	19.7%	29.7%	12.9%	1.04	1.36
	Other Regions (N: 532)	0.6%	3.4%	3.9%	18.8%	24.6%	30.5%	18.2%	1.71	1.20
**Willingness to buy**	**Sample**	**Strongly disagree**	**Disagree**	**Somewhat disagree**	**Neither agree** **nor disagree**	**Somewhat Agree**	**Agree**	**Strongly agree**	***Mean Score***	***Std. Dev.***
		−3	−2	−1	0	1	2	3		
**WTB**: How much do you agree or disagree with the following statement: I am willing to buy Fiore Sardo PDO cheese produced using traditional methods rather than industrial methods.	Sardinia (N: 249)	0.0%	1.2%	2.0%	10.04	36.1%	32.5%	18.1%	1.51	1.04
	Other Regions (N: 532)	0.9%	1.5%	2.6%	7%	28.6%	35.3%	24.1%	1.63	1.14

Before discussing the structural model results presented in Table 5, the reliability and validity of the constructs were assessed. Cronbach’s alpha values ranged between 0.74 and 0.91 across constructs, exceeding the recommended threshold of 0.70 [27]. Standardised factor loadings varied between 0.52 and 0.89, satisfying the recommended minimum level of 0.50 [28]. Composite reliability values were all above 0.70, while Average Variance Extracted (AVE) values ranged from 0.51 to 0.78, confirming convergent validity. Discriminant validity was supported by HTMT ratios below the threshold value of 0.90 [23]. Furthermore, no multicollinearity issues emerged, as VIF values ranged from 1.00 to 2.39 for the outer model and from 1.00 to 1.70 for the inner model. Overall, the measurement model demonstrated satisfactory reliability and validity properties,

(i.e., between items) are lower than 3, [28]. Specifically, the VIF values ranged from 1.00 to 2.39 for the outer model and from 1.00 to 1.70 for the inner model.

The results of the PLS-SEM estimation are presented in Table 5. We employed the bootstrapping technique [26] with 10,000 iterations to calculate the standard errors of the estimates. The analysis found that TPB beliefs significantly influenced the attitudes, subjective norms, and perceived behavioural control constructs. Specifically, the participants’ behavioural beliefs positively impacts attitudes (β = 0.62; *p* < 0.001 Sardinia group; β = 0.66; *p* < 0.001 OIR group; β = 0.65; *p* < 0.001 pooled group of participants), normative norms exert a positive and significant effect on subjective norms (β = 0.65; *p* < 0.001 Sardinia group; β = 0.63; *p* < 0.001 OIR group; β = 0.62; *p* < 0.001 pooled group of participants), and control beliefs are positively related to perceived behavioural control (β = 0.50; *p* < 0.001 Sardinia group; β = 0.49; *p* < 0.001 OIR group; β = 0.52; *p* < 0.001 pooled group of participants). Thus, the results support the TPB model’s hypotheses H1a, H1b, and H1c.

Furthermore, attitudes (β = 0.29; *p* < 0.001 Sardinia group; β = 0.36; *p* < 0.001 OIR group; β = 0.34; *p* < 0.001 pooled group of participants), subjective norms (β = 0.26; *p* < 0.001 Sardinia group; β = 0.20; *p* < 0.001 OIR group; β = 0.17; *p* < 0.001 pooled group of participants), and perceived behavioural control (β = 0.25; *p* < 0.001 Sardinia group; β = 0.18; *p* < 0.001 OIR group; β = 0.26; *p* < 0.001 pooled group of participants) contributed positively and significantly to the willingness to buy Fiore Sardo PDO cheese. Therefore, we find support for hypotheses H2a, H2b, and H2c. Finally, WTB has a positive and significant influence on WTP (β = 0.22; *p* < 0.05 for the Sardinia group, β = 0.22; *p* < 0.001 for the OIR group, and β = 0.23; *p* < 0.001 for the pooled group of participants), thus supporting hypothesis H3.

In Table 5, we also report the *p*-values of the f^2^ statistics. The statistic quantifies how much a predictor contributes to explaining the variance of a particular dependent construct in the model. All *p*-values associated with the f^2^ are very close to or below the 5% significance level, indicating that the effects in the model are statistically significant. This suggests that the predictors make a meaningful contribution to explaining the variance in the dependent variables.

Among the sociodemographic variables (we do not report the values of the estimates in Table 5 for brevity, but they are available upon request), age has a positive and significant impact on behavioural beliefs (β = 0.135; *p* < 0.05) for the OIR sample, as well as gender (β = 0.105; *p* < 0.05). Therefore, being older and female positively influences the decision to buy and pay a premium price for Fiore Sardo PDO cheese, produced using traditional methods, among participants in the OIR group. A positive and significant impact is also observed for income on behavioural beliefs (β = 0.14, *p* < 0.001), normative beliefs (β = 0.17, *p* < 0.001), and control beliefs (β = 0.09, *p* < 0.05). Participants living in Sardinia only show a positive impact of income on the control beliefs constructs (β = 0.14; *p* < 0.05).

The goodness-of-fit test statistics, R-squares, and SRMR statistics for the Sardinia and OIR groups of participants, as well as the pooled sample, indicated a good fit, in line with all threshold values accepted in the literature. Finally, to assess out-of-sample predictive power, we used the PLS Predict procedure in SmartPLS. Most indicators showed Q^2^ values (not reported in Table 5) greater than zero, confirming the models’ meaningful predictive relevance. Only CB1 yielded a slightly negative Q^2^ value (–0.005), indicating a lack of predictive power for that indicator.

## 5. Discussion

This study examined why consumers are willing to pay a premium for Fiore Sardo PDO cheese made using traditional methods and whether such preferences can sustain rural production systems. Combining conceptually the Theory of Planned Behaviour (TPB) with economic conditions derived from a Bertrand duopoly model, our findings suggest that traditional producers can justify higher prices when consumers perceive their products as higher in quality, culturally valuable, and socially legitimate. This contributes to broader debates in rural studies concerning the survival of traditional food systems under market pressure and globalised competition. Although previous TPB studies in food consumption contexts have examined healthy diets, ethical food choices, and traditional eating patterns, e.g., [12,13], limited research has investigated how TPB constructs interact with rural heritage preservation and competition within PDO markets. Our findings, therefore, contribute to extending TPB applications into the field of cultural and territorial food systems.

Consistent with hypotheses and previous TPB-based research [11,15], behavioural, normative, and control beliefs significantly influenced attitudes, subjective norms, and perceived behavioural control, which in turn predicted the willingness to buy. Importantly, willingness to buy (WTB) translated into willingness to pay (WTP), reinforcing the interpretation of WTP as a behavioural intention rather than a purely economic response [16]. These findings confirm that traditional food purchasing is driven not only by taste or price, but also by moral beliefs about supporting local shepherds, preserving rural identity, and maintaining gastronomic heritage.

The positive willingness to pay for traditional Fiore Sardo (average premiums of €3.7 in Sardinia and €5.5 in other regions) aligns with the conditions proposed by the Bertrand model: higher perceived quality and increased marginal costs justify price differentiation. This alignment between behavioural intentions and economic theory is significant. It suggests that traditional production can remain economically viable if consumers continue to value the symbolic and material aspects of handcrafted foods. This supports earlier findings indicating that PDO schemes can safeguard both product identity and rural livelihoods. However, unlike most previous research focusing primarily on quality labels or sensory preferences, this study demonstrates that willingness to pay is also linked to support for rural cultural heritage and pastoral livelihoods [29,30,31].

The positive willingness to pay for traditional Fiore Sardo aligns not only with the conditions proposed by the Bertrand model but also with previous studies showing that consumers attribute higher economic value to products associated with authenticity, territorial identity, and artisanal production ( [29] [30]. Although the structural relationships of TPB were consistent across regions, some territorial nuances emerged. Sardinian respondents reported higher perceived behavioural control, largely due to easier access to traditional Fiore Sardo. In contrast, in other Italian regions, behavioural beliefs were more strongly influenced by age and gender, suggesting that where traditional foods are less embedded in daily life, socio-demographic characteristics play a stronger role in shaping their perceived value.

Interestingly, WTP was higher outside Sardinia. This may reflect the “familiarity paradox”: products embedded in everyday consumption may be perceived as ordinary, while for distant consumers they carry symbolic capital linked to authenticity, tradition and rural heritage [32].

The results demonstrate that consumers’ willingness to pay for traditionally produced Fiore Sardo is not only a market preference but a mechanism that sustains pastoral livelihoods and territorial identity. Traditional sheep-based cheese production in Sardinia is deeply rooted in transhumance practices, local breeds, and smallholder family farms. When consumers value authenticity, cultural heritage and artisanal methods, they indirectly support the continuity of these pastoral systems and the maintenance of rural landscapes. This strengthens the argument that willingness to pay extends beyond sensory attributes and reflects cultural and socio-economic solidarity with rural producers.

Several implications arise for rural development policy and local producer organisations. First, supporting belief-based marketing actions through communication should emphasise the preservation of livelihoods, cultural continuity, and the safeguarding of local breeds and landscapes, as these beliefs strongly influence favourable attitudes and purchasing intentions. Second, expanding distribution without losing identity is crucial, as higher WTP in non-local markets suggests opportunities for rural producers to internationalise or grow domestically, provided that quality signals and origin certification remain visible and trustworthy. In addition, willingness to pay for traditional cheese highlights potential interactions with rural tourism and short supply chains. Agritourism, on-farm cheese sales, festivals, and educational rural experiences can reinforce perceived authenticity, increase producers’ margins and strengthen territorial branding. These findings suggest that Fiore Sardo is not only a food product but a cultural asset that can anchor place-based development strategies.

These findings align with recent EU policy debates on geographical indications (GIs), particularly the 2024 reform of the EU GI system, which emphasises stronger producer governance, fair value distribution along the supply chain, and safeguarding of traditional knowledge. Our results suggest that reinforcing origin labelling, transparency, and producer recognition can translate consumer beliefs into concrete economic advantages for rural communities. Therefore, Fiore Sardo and similar PDO products can serve as strategic tools in rural development policies, especially under the EU’s CAP and Rural Development Programmes, which support short supply chains, agri-food heritage, and environmental stewardship.

Despite its contributions, the study has limitations. WTP was based on stated preferences rather than actual market behaviour, potentially introducing hypothetical bias. Future research could employ discrete choice experiments or experimental auctions. Second, TPB explains a substantial portion of behavioural variance, but complementary constructs such as place attachment, trust in institutions, environmental values, or habit could enrich the framework. Finally, while Fiore Sardo PDO cheese is a relevant case, comparative studies across different PDO products and European regions would enhance generalisability and reveal how institutional arrangements interact with consumer values.

## 6. Conclusions

This study explored whether traditionally produced Fiore Sardo PDO cheese can sustain itself economically in a market dominated by industrial producers offering lower-priced alternatives. It also investigated the psychological and cultural factors that influence consumers’ willingness to pay a premium for these products. By combining the Theory of Planned Behaviour (TPB) with an economic model of price competition, we demonstrated that traditional food systems can thrive when consumers view them as high-quality, morally valuable, and culturally significant.

The results confirmed that behavioural, normative, and control beliefs significantly influence attitudes, subjective norms, and perceived behavioural control. These factors, in turn, affect consumers’ willingness to buy and their willingness to pay a higher price for the Fiore Sardo PDO cheese made using traditional processes. The observed price premiums, especially outside Sardinia, indicate that consumers are willing to pay more for traditional production methods when they recognise their cultural and social value. This supports the conditions for Bertrand competition, where higher marginal costs and perceived quality enable traditional producers to charge higher prices. The Bertrand framework was employed as a conceptual representation of competitive dynamics between traditional and industrial producers rather than as an empirically estimated market model. Future research could integrate firm-level price and cost data to empirically test competitive interactions within PDO markets.

The study also highlights notable territorial differences. In Sardinia, traditional cheese is widely accessible and an integral part of daily life, whereas in other Italian regions, the product holds symbolic significance tied to authenticity, heritage, and pastoral culture.

This finding extends previous literature on PDO products and traditional foods, which has mainly emphasised quality differentiation and origin effects, by showing that behavioural intentions related to cultural preservation and social solidarity can also contribute to the economic sustainability of traditional production systems. Overall, the findings contribute to debates in rural studies by demonstrating that PDO-labelled traditional products are not only economic commodities but also serve as tools for supporting rural livelihoods, cultural identity, and landscape preservation. However, for these systems to remain resilient, institutional support, transparent certification, and effective communication strategies that bolster consumer confidence in authenticity and rural welfare are essential.

The study relies on stated preference data collected through a contingent valuation approach, which may be subject to hypothetical bias and social desirability effects. Respondents may overstate their willingness to pay for traditionally produced foods when cultural heritage, rural livelihoods, and ethical considerations are emphasised within the survey context. Consequently, the estimated willingness to pay premiums should not be interpreted as direct measures of actual market behaviour but rather as indicators of consumers’ stated behavioural intentions and value perceptions. Future research could employ experimental auctions, revealed-preference approaches, or field experiments to assess whether stated preferences translate into actual purchasing behaviour.

Future work should also examine genuine economic behaviour through auction or experimental market designs, compare different traditional products and regions, and explore how factors such as place attachment, trust, and environmental concerns influence purchasing decisions. Nonetheless, this research highlights a key conclusion: where culture, beliefs, and perceptions of quality converge, traditional rural food systems can endure, even within competitive modern markets.

## Figures and Tables

**Table 1:** Summary statistics of the socioeconomic variables.

Socio-Economic Variables	Response Scale	Sardinia [N = 313] (Percentage)	Other Italian Regions [N = 1339] (Percentage)
**Age**	1: 18–24 years	4.0	9.0
	2: 25–34 years	8.8	16.0
	3: 35–49 years	35.0	25.4
	4: 50–64 years	38.6	37.4
	5: More than 65 years	13.6	12.2
**Gender**	1: Female	55.8	52.6
	0: Male	44.2	47.4
**Educational level**	1: Lower than a high school diploma	10.0	8.7
	2: High school diploma	59.8	49.0
	3: University degree	30.2	42.3
**Income level (euro)**	1: Less than 20,000	30.1	16.9
	2: 20,000–39,999	47.0	49.3
	3: 40,000–59,999	18.5	21.6
	4: more than 60.000	4.4	12.2
**Number of family members**	1: 1 person	12.0	10.5
	2: 2 people	30.9	29.7
	3: 3 people	25.7	28.1
	4: 4 people	22.5	24.6
	5: 5 people	7.2	5.1
	6: more than 5 people	1.6	2.0
**Monthly Cheese spending (euro)**	1: 0–19	20.5	9.2
	2: 20–49	47.0	40.0
	3: 50–99	22.5	30.5
	4: 100–149	7.2	13.9
	5: 150–199	2.4	4.0
	6: 200–300	0.4	1.6
	7: more than 300	0	0.8
**Monthly Pecorino Cheese spending (euro)**	1: 0–19	43.7	43.4
	2: 20–49	39.0	34.6
	3: 50–99	11.2	13.2
	4: 100–149	4.8	6.2
	5: 150–199	0.9	1.2
	6: 200–300	0.4	0.8
	7: more than 300	0	0.6
**Pecorino Cheese consumption** **(days of consumption)**	1: every day	4.8	6.6
	2: more than 3 days a week	37.3	23.9
	3: 2 days a week	29.3	32.0
	4: 1 day a week	16.6	17.5
	5: 2–3 days a month	8.0	10.6
	6: 1 day a month or less	4.0	9.4

**Table 5:** The r esults of structural models.

*Indirect* *Effects*									
Path	Sardinia			Other Italian Regions			Italy		
	Parameters	R^2^	f^2^ (*p*-Val)	Parameters	R^2^	f^2^ (*p*-Val)	Parameters	R^2^	f^2^ (*p*-Val)
*Indirect effects*									
**BB** **→** **ATT**	0.613 ***	0.38	0.003	0.664 ***	0.44	0.000	0.647 ***	0.42	0.000
**NB → SN**	0.646 ***	0.42	0.000	0.617 ***	0.38	0.000	0.626 ***	0.39	0.000
**CB → PBC**	0.504 ***	0.25	0.001	0.489 ***	0.24	0.000	0.524 ***	0.27	0.000
**ATT → WTB**	0.278 ***	0.39	0.049	0.362 ***	0.38	0.005	0.341 ***	0.39	0.000
**SN → WTB**	0.255 ***		0.051	0.201 ***		0.000	0.171 ***		0.030
**PBC → WTB**	0.245 ***		0.050	0.180 ***		0.007	0.257 ***		0.003
**WTB → WT P**	0.224 **	0.05	0.050	0.224 ***	0.05	0.009	0.228 ***	0.05	0.003
*Total effects*									
**BB → WTB**	0.170 ***	-		0.240 ***	-		0.221 ***	-	
**NB → WTB**	0.255 ***	-		0.202 ***	-		0.107 ***	-	
**CB → WTB**	0.124 ***	-		0.088 ***	-		0.135 ***	-	
SMRM	0.075			0.074			0.076		

Note: Parameters are standardised. * *p* < 0.1; ** *p* < 0.05; *** *p* < 0.001.

## Data Availability

Data are available upon request from the authors.
